# Public health round-up

**DOI:** 10.2471/BLT.20.010820

**Published:** 2020-08-01

**Food insecurity**

A nurse demonstrates how to make broth to help mothers feed their children at a health centre in San Pédro, Côte d’Ivoire – where around one in five children suffer from stunting. According to a new report on food security and nutrition, in 2019 between a quarter and a third of children under five worldwide were stunted or wasted (too short or too thin).

**Figure:**
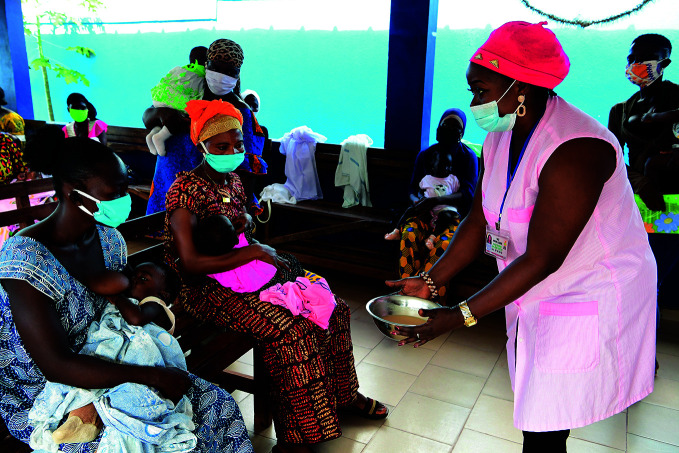
UNICEF / Frank Dejongh

## Vaccine coverage shrinks

The COVID-19 pandemic has significantly disrupted immunization programmes. Respondents from the 82 countries to an on-line ‘pulse poll’ survey conducted in June 2020 reported that disruptions to routine immunization services were widespread throughout May. Of the 61 countries reporting on the current status of vaccination coverage, 85% indicated that the level of vaccination was lower in May than in January-February 2020.

Conducted by the United Nations Children’s Fund (UNICEF), the World Health Organization (WHO), Gavi, the Vaccine Alliance (Gavi) and the Sabin Vaccine Institute, the survey confirmed the findings of an earlier pulse poll, and monthly provisional data reported to WHO between January and April. Those data indicated that as many as 1.4 million fewer doses of diphtheria-tetanus-pertussis vaccine had been administered compared to the same period in 2019. 

The reasons cited for disruption included lack of health workers and personal protective equipment. Patient concerns about exposing themselves to COVID-19 when seeking vaccination were also a factor, as were limited public transport, lockdowns and physical distancing policies.

The data were released on 15 July along with an update on worldwide immunization coverage which shows that progress on immunization coverage was stalling before the pandemic hit.

https://bit.ly/3fz7cyL

## ACT-Accelerator funding gap

The COVID-19 Tools Accelerator (ACT-Accelerator) initiative is facing a short-term funding gap of US$ 13.7 billion.

The ACT-Accelerator brings together governments, health organizations, scientists, businesses, civil society, and philanthropists, and is designed to support the development and equitable distribution of COVID-19 related tests, treatments and, vaccines.

According to costed plans for the initiative, which were included in an investment case published by WHO on 26 June, there is an immediate need for US$ 17.1 billion to fund activities over the next six months. These include research and development and the scale up of diagnostics production and distribution. Only US$ 3.4 billion has so far been pledged.

The investment case calls for a total US $ 31.3 billion in funding to achieve the goals set for diagnostics, therapeutics and vaccines by 2021. An additional US$ 27.9 billion is therefore needed. While significant, the total cost of the ACT-Accelerator's work is less than a tenth of what the International Monetary Fund estimates the global economy is losing every month due to the pandemic.

https://bit.ly/3eq4x92

## Vaccine solidarity

Seventy-five countries submitted expressions of interest to join the COVAX Facility, a mechanism designed to guarantee rapid, equitable access to COVID-19 vaccines worldwide.

According to a joint statement by Gavi and the Coalition for Epidemic Preparedness Innovations (CEPI) made on 15 July, the 75 countries would finance the vaccines from their own budgets and partner with up to 90 lower-income countries that could be supported through voluntary donations to Gavi’s COVAX Advance Market Commitment, a financing instrument aimed at incentivising vaccine manufacturers to produce sufficient quantities of eventual COVID-19 vaccines to ensure access for developing countries.

The COVAX Facility mechanism is part of the Access to COVID-19 Tools Accelerator, a global collaboration designed to accelerate the development, production, and equitable access to COVID-19 tests, treatments, and vaccines. 

https://bit.ly/32mXZpr

## Evaluating the pandemic response

An independent panel was initiated to evaluate the world’s response to the COVID-19 pandemic. The Independent Panel for Pandemic Preparedness and Response (IPPR) will be co-chaired by the former Prime Minister of New Zealand, Helen Clark, and the former President of Liberia, Ellen Johnson Sirleaf. The co-chairs will choose other panel members, as well as members of an independent secretariat to provide support.

The panel was created in response to a World Health Assembly resolution adopted in May, which calls on WHO to initiate an independent and comprehensive evaluation of the lessons learned from the world’s health response to COVID-19.

“This is a time for self-reflection, to look at the world we live in and to find ways to strengthen our collaboration as we work together to save lives and bring this pandemic under control,” said WHO Director-General Tedros Adhanom Ghebreyesus at the 9 July announcement of the IPPR’s initiation.

https://bit.ly/3folCll

## Finding the SARS-CoV-2 source

WHO experts travelled to China on 10 July to consult with Chinese scientists about progress made in understanding the origins of the novel coronavirus (SARS-CoV-2). According to a 10 July media briefing led by WHO Director-General, the consultation was to serve as the basis for a planned WHO-led international mission aimed at investigating the zoonotic source of SARS-CoV-2 and determining how it moved from an animal host to humans.

https://bit.ly/38YpM0Q

## Access to antiretrovirals threatened

Seventy-three countries reported that they are at risk of stock-outs of antiretrovirals (ARVs) for the treatment of human immunodeficiency virus (HIV) due to the COVID-19 pandemic. The reports were made in response to a WHO survey, the results of which were published on 6 July.

Among the causes cited for the disruptions were a failure of suppliers to deliver ARVs because of a pandemic-related shut-down of transport services and the impact of the pandemic on health service within countries. Twenty-four countries reported having either a critically low stock of ARVs or disruptions in supply.

The survey follows a modelling exercise conducted by WHO and the Joint United Nations Programme on HIV/AIDS (UNAIDS) in May which forecast that a six-month disruption in access to ARVs could lead to a doubling in HIV-related deaths in sub-Saharan Africa in 2020 alone.

https://bit.ly/2OkfwGC

## Medicines dropped from Solidarity Trial

The Solidarity Trial’s International Steering Committee recommended that the trial’s hydroxychloroquine and lopinavir/ritonavir arms be discontinued. WHO accepted the recommendation on 4 July.

The recommendation was made in light of evidence derived from the trial’s interim results and from a review of the evidence from all trials presented at the 1-2 July WHO Summit on COVID-19 research and innovation.

The interim results showed that the drugs produce little or no reduction in the mortality of hospitalized COVID-19 patients when compared to the standard of care.

This decision does not affect the possible evaluation in other studies of the drugs in non-hospitalized patients or as pre- or post-exposure prophylaxis for COVID-19. The interim Solidarity results are now being readied for peer-reviewed publication.

https://bit.ly/2Dza1Sd

**Cover Photo**

A girl stands in front of her tent trying not to breathe the smoke coming from the wood burning stove inside. Kashmir, Pakistan.

**Figure:**
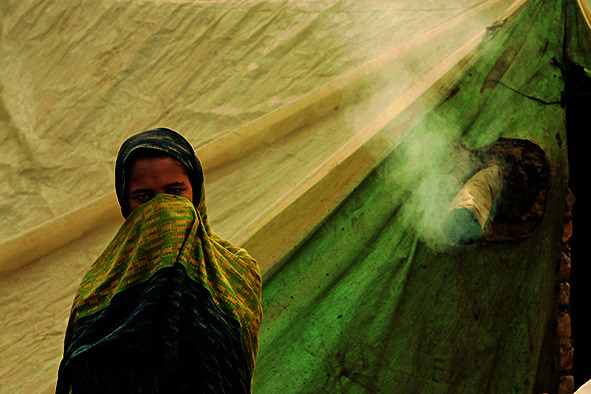
Paula Bronstein/Getty Images

## Global food insecurity

Almost 690 million people went hungry in 2019, of whom 381 million live in Asian countries, 250 million in African countries and 48 million in Latin American and the Caribbean countries.

This is according to *The state of food security and nutrition in the world*, published 13 July. The report notes that the global prevalence of undernourishment – or overall percentage of hungry people – has changed little since 2014 at around 9%, but that the absolute number of undernourished has been rising, and increased by 10 million between 2018 and 2019.

The report forecasts that the COVID-19 pandemic could result in 130 million more people being affected by chronic hunger by the end of 2020.

https://bit.ly/3fxcP0k

## Quitting tobacco with digital help

WHO launched a pilot initiative for quitting tobacco, which gives people free access to an artificial-intelligence application known as “Florence”. Florence, described as the first “digital health worker”, is available via video stream or text to help people access reliable information and develop personalized plans to quit tobacco use. Florence currently speaks only English, but will be speaking the other United Nations languages in the next three months.

The initiative will be piloted in Jordan, which has among the highest rates of tobacco use in the world and where a nation-wide ban on smoking and vaping indoors in public places was adopted at the beginning of July.

https://bit.ly/3iWluvt

## Health initiative launched to fight COVID-19

The International Olympic Committee, WHO and the United Nations launched a partnership to encourage people around the world to achieve better health as part of the COVID-19 pandemic response.

Announced on 23 June, the #HEALTHYTogether initiative aims to combat noncommunicable diseases (NCDs) through physical activity. People living with NCDs are at higher risk of severe COVID-19-related illness and death.

https://bit.ly/2WdQ35Q

**Looking ahead**

August 31 - International Overdose Awareness Day. Online events to raise awareness and reduce stigmatization. https://bit.ly/2OqOZr5

September 10 - World Suicide Prevention day, 'Working Together to Prevent Suicide.' Online events to raise awareness. https://bit.ly/30rc1E7

September 14 – 15 - WHO Regional Committee for Europe; convened online. https://bit.ly/2Cd3XP1

